# Threshold of heteroplasmic truncating MT-ATP6 mutation in reprogramming, Notch hyperactivation and motor neuron metabolism

**DOI:** 10.1093/hmg/ddab299

**Published:** 2021-10-12

**Authors:** Sebastian Kenvin, Ruben Torregrosa-Muñumer, Marco Reidelbach, Jana Pennonen, Jeremi J Turkia, Erika Rannila, Jouni Kvist, Markus T Sainio, Nadine Huber, Sanna-Kaisa Herukka, Annakaisa Haapasalo, Mari Auranen, Ras Trokovic, Vivek Sharma, Emil Ylikallio, Henna Tyynismaa

**Affiliations:** Stem Cells and Metabolism Research Program, Faculty of Medicine, University of Helsinki, Finland; Stem Cells and Metabolism Research Program, Faculty of Medicine, University of Helsinki, Finland; Department of Physics, University of Helsinki, Finland; Stem Cells and Metabolism Research Program, Faculty of Medicine, University of Helsinki, Finland; Stem Cells and Metabolism Research Program, Faculty of Medicine, University of Helsinki, Finland; Stem Cells and Metabolism Research Program, Faculty of Medicine, University of Helsinki, Finland; Stem Cells and Metabolism Research Program, Faculty of Medicine, University of Helsinki, Finland; Stem Cells and Metabolism Research Program, Faculty of Medicine, University of Helsinki, Finland; A.I. Virtanen Institute for Molecular Sciences, University of Eastern Finland, Kuopio, Finland; Department of Neurology, Kuopio University Hospital, Kuopio, Finland; Neurology, Institute of Clinical Medicine, University of Eastern Finland, Kuopio, Finland; A.I. Virtanen Institute for Molecular Sciences, University of Eastern Finland, Kuopio, Finland; Clinical Neurosciences, Neurology, Helsinki University Hospital, Finland; Stem Cells and Metabolism Research Program, Faculty of Medicine, University of Helsinki, Finland; Department of Physics, University of Helsinki, Finland; HiLIFE Institute of Biotechnology, University of Helsinki, Finland; Stem Cells and Metabolism Research Program, Faculty of Medicine, University of Helsinki, Finland; Clinical Neurosciences, Neurology, Helsinki University Hospital, Finland; Stem Cells and Metabolism Research Program, Faculty of Medicine, University of Helsinki, Finland; Department of Medical and Clinical Genetics, University of Helsinki, Finland; Neuroscience Center, HiLIFE, University of Helsinki, Finland

## Abstract

Mutations in mitochondrial DNA encoded subunit of ATP synthase, *MT-ATP6*, are frequent causes of neurological mitochondrial diseases with a range of phenotypes from Leigh syndrome and NARP to ataxias and neuropathies. Here we investigated the functional consequences of an unusual heteroplasmic truncating mutation m.9154C>T in *MT-ATP6*, which caused peripheral neuropathy, ataxia and IgA nephropathy. ATP synthase not only generates cellular ATP, but its dimerization is required for mitochondrial cristae formation. Accordingly, the *MT-ATP6* truncating mutation impaired the assembly of ATP synthase and disrupted cristae morphology, supporting our molecular dynamics simulations that predicted destabilized *a/c* subunit subcomplex. Next, we modeled the effects of the truncating mutation using patient-specific induced pluripotent stem cells. Unexpectedly, depending on mutation heteroplasmy level, the truncation showed multiple threshold effects in cellular reprogramming, neurogenesis and in metabolism of mature motor neurons (MN). Interestingly, MN differentiation beyond progenitor stage was impaired by Notch hyperactivation in the *MT-ATP6* mutant, but not by rotenone-induced inhibition of mitochondrial respiration, suggesting that altered mitochondrial morphology contributed to Notch hyperactivation. Finally, we also identified a lower mutation threshold for a metabolic shift in mature MN, affecting lactate utilization, which may be relevant for understanding the mechanisms of mitochondrial involvement in peripheral motor neuropathies. These results establish a critical and disease-relevant role for ATP synthase in human cell fate decisions and neuronal metabolism.

## Introduction

Mitochondrial DNA (mtDNA) is a maternally inherited multicopy genome coding for 13 essential polypeptides of the oxidative phosphorylation system (OXPHOS). Mutations in mtDNA are homoplasmic if present in all copies of the circular genome, or heteroplasmic if present in a proportion of genomes. Pathogenic mtDNA mutations cause a wide spectrum of primary mitochondrial diseases from distinct clinical syndromes to monosymptomatic presentations, which is partly explained by the heteroplasmy level in each tissue of a patient (1). Pathogenic mtDNA mutations show a threshold effect in regard to the detrimental level of heteroplasmy that leads to disease. The threshold varies between cell and tissue types and different mtDNA mutations, but is 60%–80% in general (2). However, the dependencies between heteroplasmy levels, biochemical or OXPHOS defects and the tissue level outcomes are not always straightforward, and involve yet uncharacterized elements.

*MT-ATP6* is one of the two mtDNA genes coding for subunits of ATP synthase or complex V of the OXPHOS, which is responsible for ATP synthesis coupled to an electrochemical gradient across the inner mitochondrial membrane in cellular respiration. Pathogenic mutations in *MT-ATP6* are typically linked to Leigh syndrome (subacute necrotizing encephalomyelopathy) (3) or the syndrome of neuropathy, ataxia and retinitis pigmentosa (NARP) (4). Descriptions of peripheral neuropathy (5) or spinocerebellar ataxia (SCA) with upper motor neuron (MN) signs (6) have extended the *MT-ATP6* associated diseases to nonsyndromic phenotypes. Indeed, recent cohort studies have concluded that *MT-ATP6* mutations cause a spectrum of mitochondrial diseases, in which neuropathy and/or ataxia are frequently observed (7–9). Renal involvement has also been described in some patients (10–12). Most *MT-ATP6* mutations are missense, but a few truncating mutations have also been described (10,13–15).

Detailed pathogenicity studies of mtDNA mutations have been complicated by the inability to introduce mtDNA mutations into cell and tissue types of interest, until recently (16). Nevertheless, reprogramming technology of somatic patient cells into induced pluripotent stem cells (iPSC) that can be differentiated into cell types of choice has provided new opportunities for experimental studies of mtDNA mutations in human cells. However, in the reprogramming process, heteroplasmic mtDNA mutations typically show a bimodal shift toward homoplasmy, either wild-type or mutant (17–21), preventing the study of intermediate heteroplasmy levels.

We studied here an unusual heteroplasmic truncating mutation in *MT-ATP6*, identified in a patient with adult-onset axonal neuropathy, ataxia and IgA nephropathy. In cellular reprogramming of patient’s skin fibroblast, instead of a bimodal shift, this severe *MT-ATP6* mutation resulted in iPSC clones having a heteroplasmy distribution between 0% and 67%. This allowed the study of *MT-ATP6* mutation effects of varying severity on cell fate decisions and in disease modeling, revealing a key disease-relevant role for ATP synthase in differentiation and metabolic regulation of human MN.

## Results

### Truncating *MT-ATP6* mutation (m.9154C>T) in adult-onset axonal neuropathy

**Figure 1:**
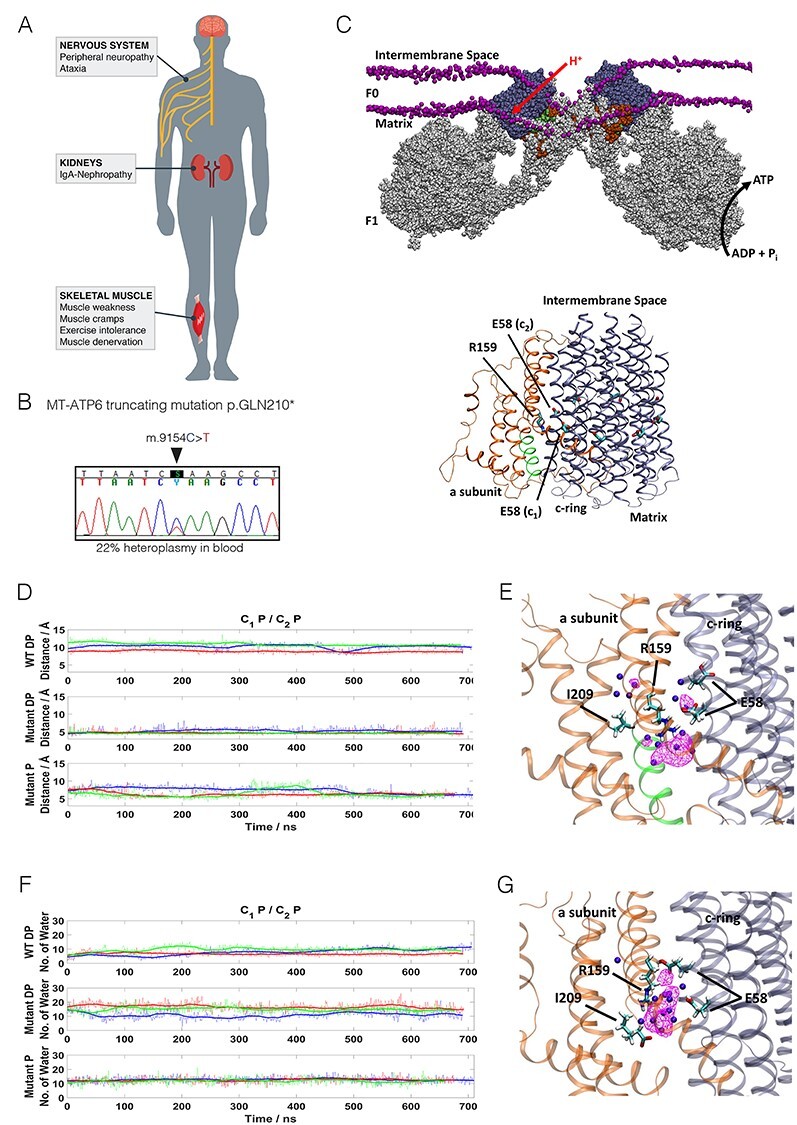
*MT-ATP6* mutation and its atomistic molecular dynamics simulations. (A) Representation of the main symptoms of the proband. (B) Sanger chromatogram showing of the m.9154C > T mutation in patient blood DNA (22% heteroplasmy). (C) Top, ATP synthase dimer model (70) embedded in a POPC lipid bilayer highly bent at the monomer–monomer interface. Lipid phosphorus atoms are shown as purple spheres. The *a* subunit and c-ring subunits are highlighted in dark orange and blue, respectively. The red arrow indicates proton transfer, and the black, ATP synthesis. Lower part, the *a* subunit (orange) and c8-ring (blue) of human ATP synthase a/c subcomplex are shown as ribbons. Arg159 of a subunit and Glu58 of c-ring are labeled. The truncated segment (Gln210-Asp224) is indicated in green. (D) Arg159 CZ—Ile209 C distance along the individual MD simulations: WT (top), mutant with deprotonated (DP) C-terminal (middle), and mutant with protonated (P) C-terminal (bottom) with all glutamates of c-ring protonated. (E) The *a/c* subcomplex of ATP synthase with all glutamates protonated (WT MD simulation). Arg159 orientates toward Glu58 of c-ring subunit c1. (F) Number of water molecules within 5 Å of Arg159 along the individual MD simulations: WT (top), mutant with DP C-terminal (middle), and mutant with P C-terminal (bottom) with all glutamates of c-ring protonated. Blue, red and green profiles in panels D and F indicate three simulation replicas with running averages (50 frames) shown in bold-thick lines. (G) The *a/c* subcomplex of ATP synthase with deprotonated C-terminal and all glutamates protonated (mutant MD simulation). Arg159 orientates toward the C-terminal of the *a* subunit. In panels E and G, Arg159 and Ile209 of the *a* subunit and Glu58 of c-ring subunits c1 and c2 are labeled. Water occupancy with isovalue 0.3 is indicated by a magenta mesh, and blue spheres depict individual water molecules.

We recently identified a truncating mutation m.9154C>T (p.Gln210*) in the *MT-ATP6* gene as part of a larger clinical exome sequencing study (22). The patient’s medical history began at age 23 when a routine urine test showed proteinuria. After a renal biopsy, he was diagnosed with IgA nephropathy (Fig. 1A), for which losartan and antiproteinuric diet were prescribed combined with yearly follow-up. He was sent for neurological assessment at age 30 because of repeated twitching and cramps of the calf muscles. The symptoms had been present for a few years, but became progressively more frequent and had started to disturb his sleep and ability to do sports.

Upon clinical examination, the patient was alert and cognitively normal. He was of muscular build, height 165 cm and weight 65 kg. He had high foot arches and symmetrical distal muscle weakness (MRC grade 4/5) in lower limbs. Proximal muscle strengths were normal. He was able to walk on toes but not on heels. He had gait ataxia and Romberg sign was positive. Increased muscle tone was noted in lower limbs. Patellar reflexes were brisk whereas Achilles tendon reflexes were absent. Plantar reflexes were indifferent. Sensation to light touch and vibration were normal. Nerve conduction study was consistent with chronic distal axonal neuropathy, which was graded moderate to severe. Needle EMG showed lower limb predominant fasciculation but no fibrillation. Brain magnetic resonance imaging (MRI) showed mild cerebellar atrophy whereas MRI of the spine was normal. Motor evoked potentials were also normal. A muscle biopsy showed neurogenic muscle atrophy with secondary myopathic changes. No cytochrome *c* oxidase negative fibers were present. A skin biopsy from the calf showed severe loss of intraepidermal small fibers. Congo staining for amyloid was negative. An ophthalmologic examination at age 33 was normal.

To the patient’s knowledge, no members in his family had been diagnosed with a chronic neurologic disease. Fluorescent restriction fragment length polymorphism (fRFLP) analysis indicated 22% heteroplasmy of the mutation in the patient’s blood (Fig. 1B).

### Atomistic molecular dynamics simulations of mutant ATP synthase *a/c* subcomplex

ATP synthase, consisting of F_1_ and F_O_ parts, catalyzes the synthesis of ATP via proton gradient across the inner mitochondrial membrane (23). Membrane-bound F_O_ part directs the energy released by the proton flux to the catalytic sites of F_1_, which protrudes into the matrix side. *MT-ATP6* encodes the *a* subunit of F_O_, which together with the *c* subunits-based rotor ring contributes to the proton translocation, as well as to biogenesis and assembly of ATP synthase (24). To understand the effects of the *MT-ATP6* m.9154C>T truncating mutation on ATP synthase function, we performed long time-scale atomistic molecular dynamics (MD) simulations of the human ATP synthase *a/c* subcomplex with the mutation (mutant) and without it (WT) (Fig. 1C). The mutant (p.Gln210*) is predicted to lack the last 17 residues of the 226 amino acid protein. We found all WT and mutant model systems to be stable throughout the MD simulations in terms of protein root mean square deviation (Supplementary Material, Fig. S1). In our WT MD simulations, positively charged Arg159 (human ATP synthase numbering) is found to orientate toward the neutral Glu58 of c-ring subunit (here c1) with an average Arg159 CZ—Glu58 CD distance of 4.75 ± 0.62 Å, which is in agreement with the structural data on ATP synthase from several different organisms (25–28). However, deletion of segment Gln210 to Thr226 brings the C-terminus of the subunit *a* inside the membrane domain, roughly one-third of the membrane thickness from the mitochondrial matrix side. As a result, the positively charged Arg159 re-orientates toward the negatively charged C-terminus of Ile209, thereby decreasing the Arg159 CZ—Ile209 C distance significantly (4.87 ± 0.59 Å, Fig. 1D, E and G). The shorter distance established between the C-terminus of the subunit and Arg159 is a clear effect of the truncating mutant in comparison to WT. Because of the localization of the C-terminus in the membrane domain, we envisaged that its charge state may be destabilized. Therefore, we simulated a neutral state of the C-terminus at position Ile209 and found that the Arg159 CZ—Ile209 C distance is still decreased, though not as much (6.83 ± 1.24 Å) and not as stably as in the case of anionic state of C-terminus, where charge–charge interaction stabilized ion-pairing between the two moieties. These data suggest that regardless of the protonation state of C-terminus, the position and dynamics of functionally critical Arg159 is distinctively perturbed from the WT, and would cause destabilization of the *a/c* subcomplex.

We find that due to the premature termination of the *a* subunit, significant hydration occurs in the region surrounding Arg159 (Fig. 1F). The hydration observed in mutant is much higher than in WT, partly due to the presence of charged states of residues (Fig. 1E, F and G). Interestingly, a higher level of hydration also occurs even if neutral state of C-terminus is simulated, because water molecules rapidly diffuse into the space created upon truncation from the matrix side, thus perturbing the local structural dynamics. Such higher level of hydration will reduce the energy barrier of proton transfer from intermembrane space to matrix (uncoupling) and cause elevated proton leak. These data bring out that both the perturbed dynamics of Arg159 as well as the excessive hydration would disrupt the function of the ATP synthase complex (see also Supplementary Material, Supplemental Information).

### Patient fibroblasts show complex V assembly intermediates and fragmented mitochondrial network

**Figure 2:**
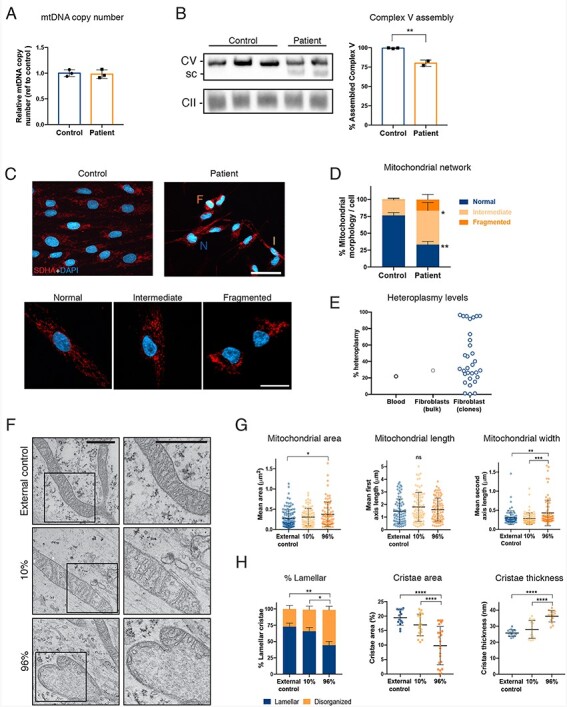
Abnormal Complex V assembly and mitochondrial morphology in patient’s skin fibroblasts. (A) mtDNA copy number determined by qPCR in unrelated healthy control and patient fibroblasts (*n* = 3 technical replicates). (B) Representative blue-native PAGE image of three control cell lines (biological replicates) and two technical replicates of patient fibroblasts. Sc denotes subcomplexes. (C) Representative immunocytochemistry images of control and patient fibroblast stained with an antibody against SDHA (mitochondria) and Dapi (nucleus). (D) Cells were manually grouped according to normal, intermediate and fragmented mitochondrial network as depicted in the lower panels (*n* = 182–204 cells from two independent experiments). N, normal; I, intermediate; F, fragmented. Scale bars 10 μm (upper panels) and 20 μm (lower panels). (E) Quantification of percent m.9154C > T heteroplasmy in patient blood, bulk fibroblasts and individual fibroblast clones by fRFLP analysis. (F) Representative electron micrographs of unrelated healthy control and patient fibroblasts with 10% or 96% mutation load. Right panels are a close up depicting the black squares on the left panels. Scale bar 500 nm. (G) Mitochondrial morphology parameters area, length and width extracted from the electron micrographs (*n* = 76–85 mitochondria per cell line). (H) Mitochondrial cristae parameters area and thickness extracted from the electron micrographs (*n* = 14–22 mitochondria per cell line). The % lamellar indicates the proportion of mitochondria with organized cristae (*n* = 76–85 mitochondria per cell line). Data shown as mean ± standard deviation; ns, non-significant, ^*^P < 0.05, ^**^P < 0.01, ^***^P < 0.001, ^****^P < 0.0001, by One-Way ANOVA followed by Tukey’s multiple comparison post-hoc test (for G and H) or Student’s *t* test (for D).

Skin fibroblasts from the patient were available for study, and showed 30% mutation heteroplasmy by fRFLP. Copy number of mtDNA was normal (Fig. 2A). Blue-native PAGE revealed an additional band for the complex V (Fig. 2B). These intermediates were detected using an antibody to the ATP synthase F_1_ subunit alpha (ATP5A), which suggests an accumulation of F_1_ subcomplexes (10). Still, about 80% of fully assembled complex V was also present in the patient cells compared to control fibroblasts.

ATP synthase F_O_ complexes form membrane-bending dimers (Fig. 1C), contributing to cristae formation and mitochondrial morphology (29). To investigate whether the mitochondrial network was affected by the *MT-ATP6* mutation in fibroblasts, we performed immunocytochemistry using an antibody against complex II subunit SDHA. Fluorescence microscopy images were classified based on the mitochondrial network into normal, intermediate and fragmented. Patient fibroblasts showed a clear shift toward mitochondrial fragmentation (Fig. 2C and D), yet around 25% of the patient cells showed a normal network. This suggested that the bulk fibroblast cultures contained cells of varying heteroplasmy levels. Thus, we subcloned 32 fibroblast lines originating from single cells and determined the heteroplasmy by fRFLP. Supporting the cell-specific effects seen on the mitochondrial network, individual clones showed heteroplasmy levels that varied between 0% and 96% (Fig. 2E). We hypothesized that the high mutant level affected cristae morphology through disassembled ATP synthase, and studied it by transmission electron microscopy. Indeed, the fibroblasts with high (96%) heteroplasmy had abnormal mitochondria and cristae (Fig. 2F). Quantification showed a significant increase in mitochondrial area and width in the 96% mutant cells as compared to 10% mutant cells (Fig. 2G), as well as more disorganized cristae that were smaller in area but thicker (Fig. 2H). These results showed that the *MT-ATP6* truncation disrupts ATP synthase assembly and mitochondrial morphology.

### Reprogramming of m.9154C>T skin fibroblasts into iPSC

**Figure 3:**
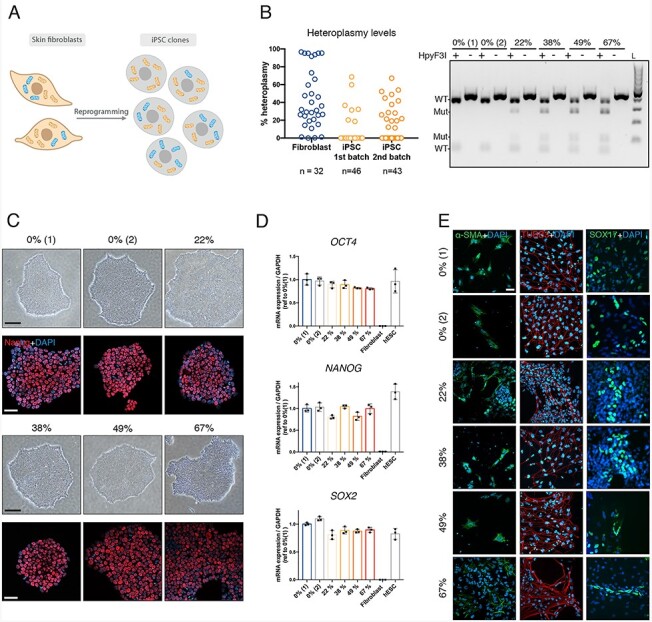
Heteroplasmy distribution of patient-derived iPSC suggest a threshold for *MT-ATP6* mutation in cellular reprogramming. (A) A schematic representation of the reprogrammed patient fibroblasts into iPSC resulting in clones with different levels of heteroplasmy ranging from 0% (isogenic control) to around 70%. (B) On the left, quantification of percent m.9154C > T heteroplasmy in individual patient fibroblast clones and two independent batches of reprogrammed iPSCs by fRFLP analysis. *n* indicates the total number of clones analyzed per batch. On the right, a representative image of an agarose gel depicting the RFLP analysis including digested (+) and non-digested (−) samples with HpyF31. L, ladder. Upon digestion, two additional bands can be observed only in the mutant clones. (C) Representative microscope images of the selected clones for follow-up experiments. Upper panel, bright field microscope image showing normal iPSC colony morphology. Scale bar 200 μm. Lower panel, immunocytochemical staining against the pluripotency marker Nanog (red) and nucleus (Dapi, blue). Scale bar 50 μm. (D) mRNA expression levels measured by qPCR of the pluripotency markers *OCT4*, *NANOG* and *SOX2* normalized to *GAPDH* of the selected clones, fibroblasts (negative control) and human embryonic stem cells (positive control), *n* = 3. Data shown as mean ± standard deviation. (E) Differentiation potential assayed by embryoid body formation followed by immunostaining against the mesoderm marker α-SMA, ectoderm marker TUBB3 and endoderm marker SOX17. Scale bar 20 μm.

Next, we reprogrammed the patient fibroblasts (bulk) into iPSC and expanded individual clones (Fig. 3A). Unexpectedly, reprogramming of patient fibroblasts with the *MT-ATP6* truncation resulted in heteroplasmy levels ranging from 0% to 67% (Fig. 3B), instead of the typically observed bimodal homoplasmy (30). Similar results were obtained in two separate batches of reprogramming. The inability to obtain iPSC clones with heteroplasmy >67% suggested a threshold for the ATP synthase defect during reprogramming.

For disease modeling the effects of the *MT-ATP6* truncation, we selected four clones with increasing levels of heteroplasmy (22%, 38%, 49% and 67%) and clones with 0% heteroplasmy as isogenic controls. Karyotypes of the clones were normal, with the exception of 22% clone, which was later found to have t (14,15) translocation (Supplementary Material, Fig. S4). Regardless of the heteroplasmy level, all clones showed normal iPSC colony morphologies and expressed similar levels of pluripotency markers *NANOG*, *OCT4* and *SOX2* (Fig. 3C and D). We also assessed the differentiation potential of each clone by formation of embryoid bodies followed by immunocytochemical analysis. All clones were positive for the endodermal marker SOX17, mesodermal marker α-SMA and ectodermal marker TUBB3 (Fig. 3E). Together, these findings demonstrate a similar pluripotency and differentiation potential in the iPSC clones with up to 67% heteroplasmy.

### m.9154C>T mutation does not affect ATP production or oxygen consumption in iPSC

**Figure 4:**
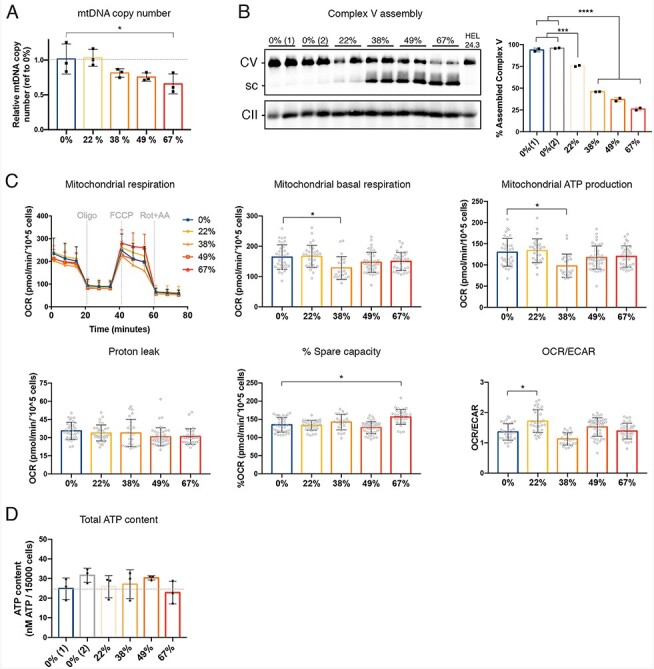
Complex V assembly defect in patient-derived iPSC is proportional to the mutation load. (A) Mitochondrial DNA copy number determined by qPCR, *n* = 3. (B) Representative Blue-Native PAGE image of two technical replicates per clone and quantification. (C) Mitochondrial oxygen consumption rate (OCR) measured with Seahorse FX analyzer. Upper panels: on the left, OCR curves depicting three basal respiration rate measurements followed by another three measurements after each injection (oligomycin, FCCP and Rotenone + antimycin). On the middle, basal mitochondrial respiration determined before oligomycin injection. On the right, mitochondrial ATP-coupled respiration calculated after inhibition of Complex V by oligomycin. Lower panels: on the left, proton leak was calculated as the remaining mitochondria respiration rate after inhibition of complex I and II by rotenone and antimycin. In the middle, % spare capacity was calculated as the difference between basal respiration and maximal respiration and expressed as %. On the right, ratio between basal mitochondrial respiration (OCR) and basal extracellular acidification rate (ECAR). *n* = 22–43, from three independent experiments. (D) Total ATP content measured using an ATPlite kit. *n* = 3 independent experiments with six wells each per cell line. Data shown as mean ± standard deviation; ns, non-significant, ^*^P < 0.05, ^***^P < 0.001, ^****^P < 0.0001, by One-Way ANOVA followed by Tukey’s multiple comparison post-hoc test or Dunnett’s test (versus 0%) for the mitochondrial respiration data.

Next, we investigated the effects of varying levels of heteroplasmy on mitochondrial function in iPSC. We observed that the mtDNA copy number decreased as the level of heteroplasmy increased (Fig. 4A). Similarly, blue-native PAGE revealed a gradual rise in the amount of F_1_ subcomplexes and a reduction in the fully assembled complex V proportionally to the heteroplasmy increase (Fig. 4B). To study the functional consequences, we analyzed the mitochondrial oxygen consumption rate (OCR) and mitochondrial ATP production of the iPSC lines using the Seahorse FX analyzer. However, the OCR and cellular ATP levels were similar in all iPSC lines (Fig. 4C), suggesting that even the 67% *MT-ATP6* mutation heteroplasmy is tolerated in the iPSC that are mainly glycolytic and characterized by having underdeveloped mitochondrial cristae (31,32).

### Threshold of *MT-ATP6* mutation heteroplasmy for MN differentiation

**Figure 5:**
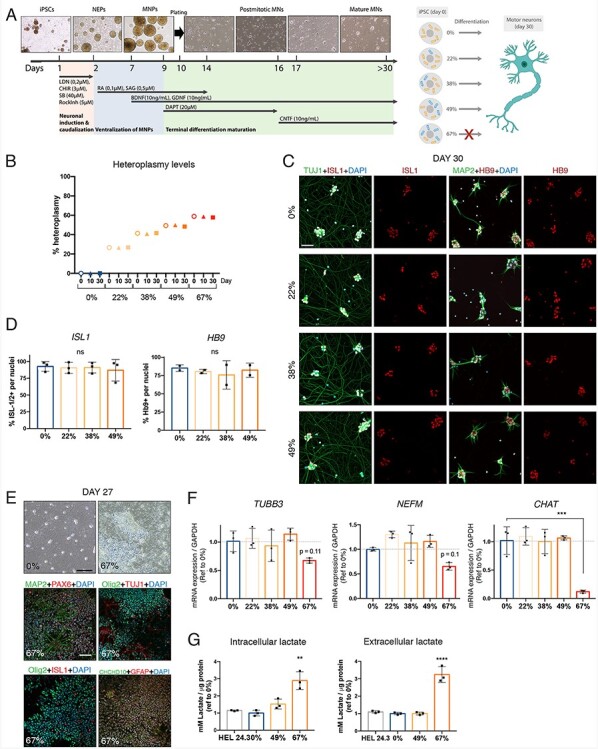
Threshold for *MT-ATP6* mutation load in MN differentiation. (A) Schematic representation of the differentiation protocol to generate iPSC-derived MN. Initially, neuronal progenitors (NEPs) are cultured in suspension for neuronal induction, caudalization and ventralization. Mature motor neuron progenitors (MNPs) are disassociated and plated on day 10 to continue growing as a monolayer. Mature MN are obtained after 30 days culture. On the right, schematic representation of the experimental design and outcome: all selected clones but the 67% mutant differentiated well. (B) Quantification of percent m.9154C > T heteroplasmy of each clone at different time points: undifferentiated iPS (0), day 10 and day 30. (C) Immunocytochemical analysis of neuronal markers TUJ1 (green), ISL1 (red), MAP2 (green) and HB9 (red) and nucleus (Dapi, blue) of neurons after 30 days of culture of cell lines 0%, 22%, 38% and 49%. Scale bar 50 μm. (D) Manual counting of ISL1- or HB9-positive nuclei relative to Dapi positive cells. Average of at least two independent experiments with *n* = 326–681 cells from 2 frames in each. (E) Upper panels, representative bright field microscope images of cell lines 0% and 67%. Cell line 67% was overgrown by dividing cells. Scale bar 200 μm. Lower panels, immunocytochemical analysis of motor neuron marker ISL1 (red), astrocyte marker GFAP (red), pan-neuron markers MAP2 (green) and TUJ1 (red), and motor neuron progenitor markers PAX6 (red) and OLIG2 (green) of neural cultures of cell lines 67% at day 27. Scale bar 50 μm. (F) Validation of neuronal markers *TUBB3*, *NEFM* and *CHAT* by qPCR and normalized to *GAPDH*, *n* = 3. (G) Intracellular and extracellular lactate levels measured using an AmpLite™ kit and normalized to protein levels, *n* = 3. Data shown as mean ± standard deviation; ns, non-significant, ^**^P < 0.01, ^***^P < 0.001, ^****^P < 0.0001, by One-Way ANOVA followed by Tukey’s multiple comparison post-hoc test.

To model the MT-ATP6 truncation in a neuronal affected cell type, we differentiated iPSC lines with different levels of heteroplasmy into spinal MN following an established 30-day differentiation protocol (33,34) (Fig. 5A). MN differentiation of 0%, 22%, 38% and 49% mutant iPSC was successful as determined by immunostaining with βIII-tubulin (TUJ1), ISL LIM homeobox 1 (ISL1), microtubule-associated protein 2 (MAP2) and MN and pancreas homeobox protein 1 (Hb9) (Fig. 5C). Manual cell count showed between 95% and 100% of the Dapi positive cells being positive for ISL1, and 85%–90% for Hb9 (Fig. 5D). Similarly, mRNA expression levels of *TUBB3*, *NEFM* and *CHAT* were comparable in the 0%–49% mutant MN (Fig. 5F). However, the 67% mutant iPSC were unable to differentiate into a pure MN culture, but instead showed a dramatic accumulation of proliferating cells starting from day 14 when the cells should become post-mitotic. By day 27 the dish was almost fully covered with proliferating cells (Fig. 5E). To elucidate the nature of these proliferating cells, we used immunostaining with antibodies targeted to different cell types, including MN (ISL1), astrocyte (GFAP), pan-neuron (MAP2, TUJ1 and NEFM) and MN progenitor (PAX6, Olig2) markers (Fig. 5E), and found positivity for all of these markers in the 67% culture. Additionally, the mRNA levels of neuronal markers, in particular the mature MN marker *CHAT* was low in the 67% culture (Fig. 5F). The high cell proliferation in 67% cultures resulted in a clear pH change of the media starting at day 14. Indeed, intra- and extracellular lactate as well as total ATP content were highly elevated at days 14 and 16 (Fig. 5G and Supplementary Material, Fig. S5). To test if heteroplasmy levels were preserved during differentiation, as has been previously reported (21,35), we performed fRFLP analysis from samples collected on days 0, 10 and 30 of differentiation (Fig. 5B), and found that the heteroplasmy levels stayed remarkably constant during the differentiation protocol.

### High heteroplasmy of MT-ATP6 truncation hyperactivated Notch

**Figure 6:**
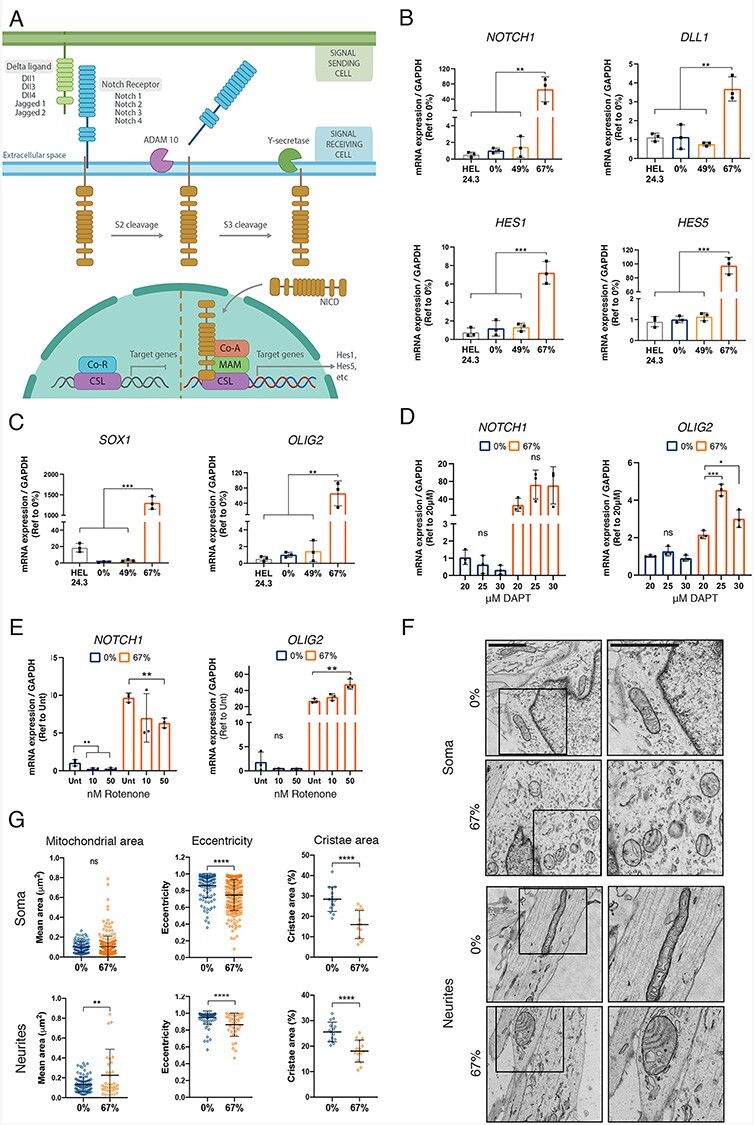
Hyperactivation of the Notch pathway prevents differentiation into MN. (A) Schematic representation of the Notch signaling pathway. Maturation of the Notch receptor is initiated upon interaction between a Notch ligand and a Notch receptor in the extracellular space. After the interaction, the metalloprotease ADAM10 cleaves the outer part of the Notch receptor, and the γ-secretase cleaves the inner part, releasing the intracellular domain of the Notch protein NICD, which moves to nucleus to activate the transcription factor CSL and trigger the expression of target genes including *HES1* and *HES5*. (B and C) Expression levels determined by qPCR in mature motor neurons (day 30), *n* = 3. (B) *NOTCH1* receptor, *DLL1* ligand and two target genes *HES1* and *HES5.* (C) Early neuronal progenitor markers *SOX1* and *OLIG2*. (D and E) Expression levels of *OLIG2* and *NOTCH1* determined by qPCR in 0% and 67% mutant lines treated with increasing levels of DAPT to block the Notch response from days 10 to 16 (D) or rotenone to block respiratory chain complex I from days 10 to 20 and followed by a recovery period without the inhibitor from days 20 to 30 (E), *n* = 3 and reference to each corresponding 20 μM (DAPT) or Unt (rotenone) group. Gene expression levels were normalized to *GAPDH*. (F) Representative electron micrographs of somas and neurites from 0% and 67% mutant motor neuron progenitors at day 15. Right panels are a close up depicting the black squares on the left panels. Scale bar 500 nm. (G) Mitochondrial morphology parameters area and eccentricity (a measure of how rounded an object is, 1 = perfect line and 0 = perfect circle) extracted from electron micrographs of somas (*n* = 110–242 mitochondria) and neurites (*n* = 38–94 mitochondria); and mitochondrial cristae area per mitochondria extracted from electron micrographs from somas and neurites (*n* = 15 mitochondria). Data shown as mean ± standard deviation; ns, non-significant, ^*^P < 0.05, ^**^P < 0.01, ^***^P < 0.001, ^****^P < 0.0001, by One-Way ANOVA followed by Tukey’s multiple comparison post-hoc test for (B–E) or by Student’s *t* test (G).

Notch signaling pathway is a key regulator of glial and neural stem cell fates (36)(Fig. 6A). Notch pathway activation enhances the proliferation of neural precursors and facilitates the terminal differentiation of glial cells, whereas Notch inactivation is required for MN maturation. For this reason, DAPT, an inhibitor of the γ-secretase that cleaves the Notch receptor for the pathway activation, is used in the MN differentiation protocol between days 9 and 16. We hypothesized that despite DAPT, Notch signaling was activated and impaired MN differentiation of the 67% *MT-ATP6* mutant iPSC.

By qPCR analysis of Notch target genes *HES1* and *HES5*, and other Notch pathway markers, we identified that Notch was indeed activated in 30-day MN cultures of the 67% *MT-ATP6* mutant. We found high expression levels of the Notch receptor *NOTCH1*, ligand *DLL1*, and target genes *HES1* and *HES5* (Fig. 6B)*,* as well as early neuron progenitor markers *SOX1* and *OLIG2* (Fig. 6C)*.* Conversely, the expression of the mature MN marker *CHAT* was significantly lower in the 67% mutant (Fig. 5F). These results indicated that the Notch signaling pathway was hyperactivated in 67% *MT-ATP6* mutant cells that were unable to differentiate beyond MN progenitor stage. Furthermore, increasing the dose of DAPT did not rescue the differentiation defect as the expression levels of *NOTCH1* and *OLIG2* did not diminish in the 67% *MT-ATP6* mutant iPSC (Fig. 6D).

Inhibition of OXPHOS complex I by rotenone in 3D neural cultures was recently reported to hyperactivate Notch (37). However, in our MN differentiation, neither the inhibition of complex I by rotenone nor the inhibition of ATP synthase by oligomycin activated Notch signaling at the level of mRNA expression or induced progenitor proliferation in the control cells similarly to the 67% *MT-ATP6* mutant (Fig. 6E and Supplementary Material, Fig. S6).

Finally, we studied if mitochondrial ultrastructure was altered in the 67% *MT-ATP6* mutant neuron progenitors (day 15), and found more rounded mitochondria in mutant cell somas with a lower cristae density (Fig. 6F and G and Supplementary Material, Fig. S7). Some cells in the progenitor culture had neurites, and mitochondria in the mutant neurites were also round whereas they were elongated in control cell neurites. These results suggested that the role of ATP synthase assembly in the regulation of mitochondrial morphology is critical for MN differentiation.

### Lower heteroplasmy *MT-ATP6* mutant causes a metabolic shift in mature MN

**Figure 7:**
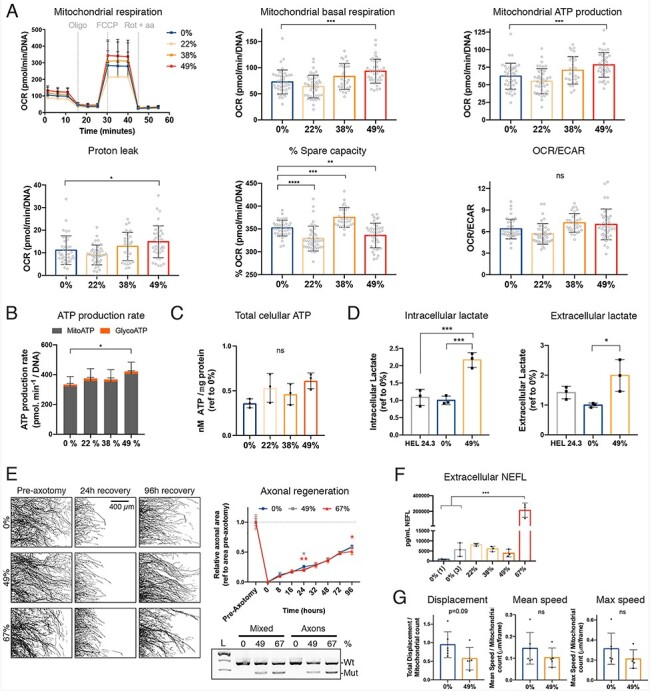
iPSC-derived MN with 49% heteroplasmy have altered mitochondrial respiration and increased lactate. (A) OCR measured with Seahorse FX analyzer as in Figure 4C, *n* = 29–41, from three independent experiments. (B) Mitochondrial and glycolytic ATP production rate measured with Seahorse FX analyzer, *n* = 15. (C) Total ATP content measured using an ATPlite kit. *n* = 3 independent experiments with six wells each per cell line. (D) Intracellular and extracellular lactate levels measured using an AmpLite™ kit and normalized to protein levels, *n* = 3. (E) Axonal regeneration capacity using microfluidic devices. Axotomy was performed at day 21. Left panels are representative masks of the axonal compartment before axotomy, 24 h and 96 h after recovery. Scale bar 400 μm. Right upper panel, quantification of axonal area during the indicated time points. *n* = 5 images per cell line from one device. Right lower panel, representative gel of fRFLP analysis showing heteroplasmy levels of each clone in the soma-dendritic compartment and axonal compartment. (F) Extracellular levels of NEFL measured from culture media collected at day 30, *n* = 3. (G) Mitochondrial axonal trafficking at day 25. Mitochondrial axonal transport parameters were extracted from five movies from one device. Data shown as mean ± standard deviation ns, non-significant, ^*^P < 0.05, ^**^P < 0.01, ^***^P < 0.001, ^****^P < 0.0001, by One-Way ANOVA followed by Tukey’s multiple comparison post-hoc test, Dunnett’s test (versus 0%) for the mitochondrial respiration data, and Student’s *t* test for axonal transport.

Next, we investigated whether MN with heteroplasmy levels below 67% could be used in disease modeling of peripheral neuropathy. We analyzed the OCR of MN derived from iPSC with 0%, 22%, 38% and 49% heteroplasmy using the Seahorse FX analyzer (Fig. 7A and B). We found that the mitochondria in 49% mutant MN had higher respiration and produced more mitochondrial ATP at the expense of reduced spare capacity compared with the isogenic 0% MN, and had a significant increase in proton leakage. MN mainly use mitochondrial oxidation, and the OCR/ECAR ratio was comparable in all clones (Fig. 7A) as well as cellular ATP content (Fig. 7C and Supplementary Material, Fig. S8A and B). However, interestingly, lactate content was doubled in 49% mutant MN compared to controls (Fig. 7D), indicating a metabolic shift.

Next, we tested if the *MT-ATP6* mutation affected axon growth and regeneration. We differentiated MN in microfluidic devices with two compartments to separate the somas and neurites (mixed) from axonal compartment. Unexpectedly, the 67% mutant was also able to grow axons, although its mixed compartment was full of progenitors (Fig. 7E, left panel). We tested if mitochondria containing lower mutant levels were transported to the axons, but did not identify differences in heteroplasmy levels between compartments (Fig. 7E, lower right panel). We performed an axotomy at day 21 on the axonal compartment to trim the axons and followed the axonal regeneration for 4 days, but did not identify consistent impairment of axon regeneration speed in mutant MN (Fig. 7E, upper right panel). Axon shape was, however, abnormal in the 67% mutant MN (Fig. 7E, left panel), and as an indication of axon degeneration, we found extremely high levels of extracellular neurofilament light (NEFL) in the 67% mutant cultures (Fig. 7F).

We also stained mitochondria with MitoTracker prior to live imaging in microfluidic devices and analyzed videos for mitochondrial displacement and speed. Both parameters were slightly but not significantly reduced in 49% mutant MN (Fig. 7G). In conclusion, iPSC with 49% mutation load differentiated normally into MN, but showed altered mitochondrial metabolism supporting their use as disease models for axonal neuropathies.

## Discussion

ATP synthase is of key importance for cellular energy metabolism, yet its defects of varying severity cause human disease. Importantly, ATP synthase is not only required for ATP production but also has a major role in regulating mitochondrial morphology by modulating cristae shape (38,39). We studied here a severe truncating mutation in *MT-ATP6*, a mitochondrial subunit of ATP synthase. This heteroplasmic mtDNA mutation was present at the level of 22% in the patient’s blood, and caused axonal neuropathy with onset in adulthood. According to our MD simulations, the truncation is expected to perturb ATP synthase function and destabilize the *a/c* subcomplex. This disrupts the assembly of the complex, and in turn, cristae morphology, which we verified in patient cells. Our results with patient-derived iPSC carrying varying heteroplasmy levels of the mutation highlight the key role of ATP synthase in cell fate decisions and neuronal metabolism. They further illustrate the significance of understanding the functional consequences of threshold effects of mtDNA mutations in tissue-specific human diseases. We suggest that the regulation of mitochondrial morphology by ATP synthase dimers contributes to the observed threshold effects.

Using patient-derived cell models, we demonstrated cell type-specific dependencies for ATP synthase function. Indicative of the differential metabolic and energy requirements of cell types, skin fibroblasts grown in glucose medium were viable and proliferating even with near homoplasmic mutation levels, whereas we could not obtain iPSC with a higher mutation load than 67%, although iPSC are known to be mostly glycolytic. In fact, instead of ATP production, stem cell maintenance may prefer mitochondria for anabolic reactions (40), which in turn may be limited by compromised mitochondrial morphology. The iPSC with 67% heteroplasmy proliferated well and differentiated into MN progenitors, but then had a block in differentiation into MN. Finally, in mature MN, mitochondrial respiration was normal in 38% heteroplasmy, but no longer in 49% heteroplasmy. The increased mitochondrial respiration in the 49% mutant might reflect an increased energy demand or, since the proton leak is also significantly increase in these cells, it might indicate a compensation mechanism to maintain the correct proton gradient across the mitochondrial inner membrane. These observed threshold effects in our cell models are likely to be more dramatic than those of the previously iPSC-modeled missense *MT-ATP6* mutations (35,41,42), because of the C-terminal truncation that severely compromises ATP synthase function. Although not directly tested here, we speculate that the truncating mutation is tolerated in human tissues at a lower heteroplasmy level than most pathogenic mtDNA mutations.

We showed that the pathogenic *MT-ATP6* truncation has a threshold for hyperactivation of Notch, which was accompanied by changes in mitochondrial ultrastructure, whereas inhibition of OXPHOS by rotenone or oligomycin did not activate Notch or induce progenitor proliferation. This suggested that the Notch hyperactivation resulted from deficient assembly of the ATP synthase that affected mitochondrial morphology and ultrastructure. In line with this interpretation, ATP synthase-dependent crista maturation was previously found to be a key developmental process required for Drosophila germ line differentiation independent of OXPHOS (43). Furthermore, mitochondrial fusion defects have been shown to inhibit cardiomyocyte differentiation via Notch signaling (44), and a positive feedback loop between Drp1-mediated mitochondrial fission and Notch signaling pathway has been proposed (45). Hyperactive Notch phenotype could not be overcome in the *MT-ATP6* mutant by increasing the dose of DAPT, an inhibitor of Notch, which was also the case for the mitochondrial fusion deficient cardiomyocytes (44). In contrast, a recent study reported Notch activation in neural MELAS organoids (37). However, in that models Notch was suppressed by DAPT, which was not normally used in the differentiation protocol. Based on our results and studies reported by others we suggest that mitochondrial structural changes caused by the pathogenic ATP synthase defect are a strong driver for Notch hyperactivation.

Curiously, the patient studied here also had IgA nephropathy, which has also previously been linked to *MT-ATP6* mutations. IgA nephropathy occurs when IgA deposits build up in the kidneys, causing inflammation, and the cause is typically not known. Previous study of a patient with severe *MT-ATP6* missense mutation and IgA nephropathy found strong mitochondrial fragmentation in a renal biopsy (12). Interestingly, Notch activation has been reported in renal tissue of patients with IgA nephropathy (46). Therefore, we speculate that ATP synthase associated mitochondrial morphology changes may also predispose to IgA nephropathy.

Mitochondria are critical for the maintenance of long axons of peripheral nerves, which is demonstrated by hereditary axonal neuropathies being caused by mutations in genes that regulate mitochondrial form and dynamics (47,48). Thus, our result that neuropathy-causing *MT-ATP6* mutation affects the assembly of ATP synthase is consistent with impaired dynamics as a possible disease mechanism. In the mutant cultured MN, we did not observe obvious changes in the distribution of mitochondria in the axons or in mitochondrial movement. Given the high impact that mitochondrial morphology has on neuronal differentiation, mitochondrial ultrastructure changes could likewise impact the metabolic needs of peripheral axons, even if the mitochondria are normally transported. Interestingly, our MD simulations indicated that the truncating *MT-ATP6* mutation would significantly increase hydration in the region surrounding Arg159, which will reduce the energy barrier of proton transfer from intermembrane space to matrix (uncoupling) and elevate proton leak. In line, we identified a threshold for the *MT-ATP6* mutation (49%) that increased mitochondrial respiration and proton leakage in mature MN, partially with the expense of mitochondrial spare capacity. Simultaneously, intracellular and extracellular lactate levels were increased, suggesting a shift in MN metabolism. The definitive roles of neuronal lactate are not fully proven (49), however, a recent study using the same iPSC-MN differentiation protocol as used by us here identified that lactate oxidation increased upon differentiation into MNs (50). Thus, it is possible that the ATP synthase deficient MN rewire their metabolism for enhanced use of lactate in energy production. More detailed characterization of lactate rewiring is needed to understand its role in MN metabolism and in the pathogenesis of axonal neuropathies.

In conclusion, patient-derived iPSC with varying levels of mtDNA mutation heteroplasmy provide new models for studying threshold effects in cell differentiation and function. The severe *MT-ATP6* mutation studied here highlights the neuronal role and requirement for ATP synthase. Furthermore, differentiation of these iPSC lines into various other cell types would be of interest to elucidate the cell type-specific dependencies on ATP synthase function.

## Materials and Methods

### Cell lines

This study involves human iPSC and skin fibroblasts derived from healthy donors and a patient (*MT-ATP6* mutation) after providing written consent for the use of patient data and material. Human patient stem cell research was approved by the Coordinating Ethics Committee of the Helsinki and Uusimaa Hospital District (Nr 95/13/03/00/15).The *MT-ATP6* mutation was corroborated by Sanger sequencing of DNA and cDNA from patient’s skin fibroblasts using the primers indicated in Supplementary Material, Table S2.

### Atomistic MD simulations

We performed atomistic MD simulations of small model systems (~130 000 atoms) representing the *a* subunit and c-ring (8 c subunits) of human F_o_ ATP synthase (Supplementary Material, Table S1). To this day no three-dimensional atomic structures of human ATP synthase have been solved. Therefore, we constructed a homology model of human a/c subcomplex based on *Sus scrofa* ATP synthase structure (6J5I (26), resolution of 3.34 Å), which has sequence similarities of 86% and 92% for the *a* and *c* subunits, respectively. To represent the human patient-derived ATP synthase mutant, we deleted residues Gln210 to Thr226 from the *a* subunit of the constructed WT model. The WT and mutant ATP synthase models were embedded in a lipid bilayer of POPC lipids, 163 (165 for mutant) and 171 lipids in the lower and upper leaflet, respectively, including six uniformly distributed lipids inside the c-ring, and solvated in TIP3P water with 0.15 M NaCl using CHARMM-GUI and associated tools (51). Residues Glu145 and Glu203 of *a* subunit and Glu58 of the eight c-ring subunits were patched neutral, unless stated otherwise (see Supplementary Material, Table S1), while standard protonation states were considered for all other charged residues. N- and C-terminals of the protein were treated by the CHARMM NTER and CTER (or CNEU for the *a* subunit) patches, and CHARMM force field was used for the protein, membrane, water and ions (52,53). All model systems were subjected to an energy minimization using Gromacs 2019.5 software (54) for 5000 steps, with a maximal force <1000 kJ mol^−1^ nm^−1^ by applying the steepest decent algorithm. Position restraints of 4000, 2000 and 1000 kJ mol^−1^ nm^−2^ for backbone and sidechain atoms of the protein and the phosphate atom of the lipids, respectively, were applied together with the dihedral angle restraints of 1000 kJ mol^−1^ rad^−2^ for the dihedral angles spanned by the lipid atoms C3-C1-C2-O21 and C28-C29-C210-C211. The minimized structures were subjected to seven equilibration runs of 25, 25, 25, 100, 100, 100 and 1000 ps with decreasing position restraints using the Berendsen thermostat and barostat (55) in the first six runs. For the final equilibration run and production runs (see Supplementary Material, Table S1 for individual simulation length) Parrinello–Rahman barostat (56) and Nosé–Hoover thermostat (57,58) were applied.

### Cell culture and reprogramming

All cells used in this study were cultured at 37°C, in a humidified atmosphere, normoxia and 5% CO_2_. Skin fibroblasts were maintained on DMEM High-Glucose (Lonza #12-614F) supplemented with 2 mM L-Glutamate (Life Technologies #250300081), 10% FBS (Life Technologies #10270106), 1× penicillin/streptomycin (Life Technologies #15140122) and 50 μg/ml Uridine (Sigma #U3003). Skin fibroblasts from a healthy individual of the same sex and age as the patient were used as a control. Single-cell fibroblast clones were obtained by seeding 0.5 cells/per well in a 96 well plate and expanded accordingly. Skin fibroblasts from the patient were reprogrammed into pluripotent stem cells via episomal plasmid vectors at Biomedicum Stem Cell Center (University of Helsinki, Finland), as previously described (59). iPSC were maintained on Matrigel-coated (Corning) plates with E8-medium (Gibco) supplemented with E8-supplement (Gibco), 50 μg/ml Uridine (Sigma #U3003) and 100 μg/ml Primocin™ (InvivoGen). iPSC were passaged with 0.5 mM EDTA (Invitrogen) in PBS when 70% confluent. iPSC clones with different levels of heteroplasmy were selected for further experiments: 0% (used as isogenic control), 22%, 38%, 49% and 67%. When indicated, a validated control iPSC cell line, HEL24.3, was used as an external control (60). iPSC experiments were done with cells from passage numbers between 19 and 30, and passage 44 for the external iPSC control.

### MN differentiation

iPSC were differentiated into MN following a protocol described elsewhere (33,34) with slight modifications. Neuronal basal medium (vol:vol DMEM/F-12 (Gibco #31331-028) and Neurobasal®(Gibco #21103-049)) was supplemented with N2 (Life Technologies), B-27 (Life Technologies), 0.1 mM L-ascorbic acid (Santa Cruz), 50 μg/ml Uridine (Sigma #U3003) and 100 μg/ml primocin (InvivoGen). For neural induction and caudalization to obtain neuroepithelial progenitor (NEP) cells, iPSCs were dissociated using EDTA and 100 000 cells/cm^2^ were plated on ultralow attachment dishes. On day 0, neuronal basal medium was supplemented with 3 μM Chir-99 021 (Selleckchem), 40 μM SB431542 (Millipore), 0.2 μM LDN-193189 (Sigma) and 5 μM Y-27632 (Selleckhem). The medium was replaced the following day. From day 2 to 6, media was changed to neuronal basal medium supplemented with 0.1 μM retinoic acid (ThermoFisher) and 0.5 μM SAG (Millipore). On day 7, medium was replaced with neuronal basal medium supplemented with retinoic acid and SAG as previously, plus 10 ng/ml BDNF (Peprotech) and GDNF 10 ng/ml (Peprotech). On day 9, medium was replaced as previously, and 20 μM DAPT was added to the fresh media. On day 10, the MN spheres were dissociated into single cells with Accumax (Invitrogen), and MN progenitors were plated on 50 μg/ml poly-D-lysine (Merck Millipore) and laminin 10 μg/ml (Sigma–Aldrich) coated plates at 50 000 cells/cm^2^. On day 11, fresh medium was added to the wells. On day 14, half of the medium was replaced with neuronal basal medium supplemented only with DAPT, BDNF and GDNF. From day 16 on, we used neuronal basal medium supplemented with BDNF, GDNF and CNTF (each 10 ng/ml, Peprotech) and replaced half of the culture medium every other/third day. Unless otherwise indicated, samples were collected on day 30.

### Embryoid body formation

iPSC were cultured on matrigel and Essential 8 (Gibco) until they reached 80% confluence. Then, cells were detached using 0.5 mM EDTA in PBS and the colonies were disassociated into small clusters of cells. The cell clusters were transferred into ultralow attachment plates (Corning, 3471) and cultured on Essential 6 (Gibco). Media was supplemented with 10 μM ROCK inhibitor (Y-27632 2HCl, Seleckchem, #S1049) only for the first two days, and then changed regularly every two days. After two weeks, the spheroids were plated on gelatin-coated (0.1%, Sigma, G1890) dishes with cover glasses, and cultured for an additional week. Then, samples were immunocytochemically stained as described below.

### Immunocytochemistry

iPSC, embryoid bodies and MN were cultured on cover glasses and fixed with 4% paraformaldehyde (PFA) for 15 min at RT and then permeabilized with PBS containing 0.2% Triton X-100 (Fisher) for 10 min. Cells were blocked with 5% protease-free BSA (Jackson Immuno Research) in 0.1% Tween20 in PBS-T for 2 h in RT. Cells were incubated overnight at +4°C in blocking buffer containing 5% bovine serum and primary antibodies. Next day, cells were washed with PBS-T and then incubated with secondary antibody for 1 h at RT. Cover glasses were applied on microscope slides with Vectashield DAPI (#H-1200) and imaged with Axio Observer Z1 (Zeiss) inverted fluorescence microscope. The antibodies used are listed in Supplementary Material, Table S3. For mitochondrial network analysis in fibroblasts, the numbers of cells with normal, intermediate or fragmented mitochondrial network were counted manually.

### Transmission electron microscopy

Cells were cultured on cover slips and fixed in 0.1 M NaCac buffer containing 2% glutaraldehyde for 30 min at room temperature. Fixed cells were processed by standard methods and imaged with Jeol JEM-1400 transmission electron microscopy (TEM) (Jeol Ltd, Tokyo, Japan) operating at 80 kV. Mitochondrial parameters were analyzed using Microscopy Image Browser (61).

### RNA extraction and RT-qPCR analysis

RNA was extracted using NucleoSpin RNA extraction kit (Macherey-Nagel) and quantified using NanoDrop™1000 spectrophotometer (Thermo Fisher). Forty nanograms of RNA was reverse transcribed using Maxima first strand cDNA synthesis kit (Thermo Fisher), and 5 ng of cDNA were analyzed by qRT-PCR amplification in CFX Real-time system C1000T (Bio-Rad) with SYBR-green Flash (Thermo Fisher). Two technical replicates per sample were measured. Relative mRNA expression was calculated using the ΔΔCt method and normalized to *GAPDH* (for nuclear transcripts). The primers used are indicated in Supplementary Material, Table S2.

### Blue-native PAGE

The assembly of mitochondrial respiratory chain complexes was evaluated with blue-native PAGE using isolated mitochondria from iPSC and fibroblasts as described elsewhere (62), followed by western blot analysis of mitochondrial Complex V ATP5 and Complex II SDHA. Antibodies used are indicated in Supplementary Material, Table S3. Detection was done by Pierce™ ECL Western Blotting Substrate (ThermoFisher Scientific) and imaging with Molecular Imager ChemiDoc XRS+ with ImageLab (Bio-Rad). The % of assembled Complex V was calculated as the intensity of the upper band (CV) divided by the total (CV + subcomplexes) and multiplied by 100.

### mtDNA copy number measurement

Total cellular DNA was isolated using proteinase K and SDS lysis followed by phenol:chloroform extraction and ethanol precipitation (63). In order to increase the efficiency of the PCR reaction and prevent variations caused by different DNA topologies, the DNA samples were gently sonicated using an ultrasonic processor (VibraCell, TAMRO) set at 50% duty cycle and total DNA was measured using Qubit Fluorometer (Thermo Fisher). MtDNA copy number was quantified as described elsewhere (64). Levels of mitochondrial (*MT-*ND5 or *MT-TL1*) and nuclear (*B2M* or *ACTB*) DNA were analyzed by qPCR amplification in CFX Real-time system C1000Touch (Bio-Rad) with SYBR-green Flash (Thermo Fisher) using 0.5 μM per primer and the following thermoprofile: 7 min at 95°C followed by 40 cycles of a two-step amplification: denaturation at 95°C for 10 s and annealing and elongation at 60°C for 30 s. Two technical replicates per sample were measured. The primers used are indicated in Supplementary Material, Table S2.

### DNA restriction fRFLP

Heteroplasmy levels in patient fibroblasts, iPSC clones and MN were assayed by fRFLP. Briefly DNA was extracted from frozen pellets using NucleoSpin DNA extraction kit (Macherey-Nagel) and quantified with DeNovixTMDS-11 FX spectrophotometer. Next, 60 ng of DNA was amplified in a PCR reaction with MyTaq HS Red Mix (Bioline) using 0.375 μM per primer and the following thermo profile: 1 min at 95°C followed by 40 cycles of 3-step amplification: 15 s at 95°C, 15 s at 63°C and 10 s at 72°C. For the last cycle, FAM labeled *MT-ATP6* reverse primer was added at 0.35 μM, followed by incubation for 5 min at 72°C. Next, samples were digested with FastDigestHpyF3I restriction enzyme (ThermoFisher). Half of each sample was separated on standard 2% agarose gel with SYBR™safe DNA gel stain at 110 V for 60 min to check the quality of the samples and confirm the restriction. After this, the remaining half of each sample was run for 1 bp resolution electrophoresis with ABI3730xl DNA analyzer (Applied Biosystems) at the Finnish Institute of Molecular Medicine. The results were analyzed via GeneMapper software (ThermoFisher). The primers used are indicated in Supplementary Material, Table S2.

### OCR and ATP production rates

Mitochondrial OCR in iPSC and MN was determined using Seahorse XF96 Extracellular Flux Analyzer (Agilent) as described elsewhere (65). Briefly, iPSCs were seeded at density of 25 000 cells/well on Matrigel-coated (Corning) XF96 Seahorse plates in the presence of 10 μM Y-27632 (Selleckchem) the day before the experiment (66). Next day, the medium was replaced 1 h before the measurement with Seahorse XF Base Medium (Agilent #103335–100) supplemented with 1 mM pyruvate, 2 mM glutamine, 25 mM glucose and 5 mM HEPES and pH adjusted to 7.4. The OCR was measured three times in baseline conditions and three times after each injection: 1.5 μM oligomycin (Sigma), 0.25 μM uncoupler carbonilcyanide p-triflouromethoxyphenylhydrazone (FCCP) (Sigma) and 1 μM rotenone (Sigma) and antimycin-A (Sigma). OCR data were normalized to cell number by staining iPSCs with Hoechst and imaging with Cytation5 plate reader. For MN, on day 10 of the differentiation 20 000 cells were seeded on poly-D-lysine and laminin coated XF96 Seahorse plates, and cells were maintained up to day 30 following the MN differentiation protocol. At day 30, OCR was measured as for iPSC but using 2 μM oligomycin, 5 μM FCCP and 1 μM rotenone and antimycin-A. OCR data were normalize to DNA concentration using CyQUANT kit (Invitrogen). At least three independent runs were performed (only two for 0% (2) clone iPSC), with each run containing 12 wells (iPSC) or 18 wells (MN) per cell line. Only wells with a homogeneous monolayer of iPSC or MN were included in the analysis. Glycolytic and mitochondrial ATP production rate were determined with Seahorse XF96 Extracellular Flux Analyzer (Agilent) using the same media as before. The OCR was measured three times in baseline conditions and three times after each injection: 1.5 μM oligomycin (Sigma) and 1 μM rotenone (Sigma) and antimycin-A (Sigma). Glycolytic and mitochondrial ATP production rates calculation were determined following manufacturers´ recommendations and (67).

### ATP and lactate content

Total cellular ATP content was measured using an ATPlite assay kit (PerkinElmer) according to manufacturer’s instructions. Briefly, 15 000 cells were counted and used to evaluate ATP content in iPSC, whereas 20 000 neuron progenitor cells were seeded in a 96-well plate and cultured as normally until day 30, when cells were lysed on the plate prior the assay. Six wells per cell line were used. Luminescence was monitored with an EnSpire (PerkinElmer) microplate reader with 0.1 s measurement time and normalized to the cell count (iPSC) or protein levels (MN). Intracellular and extracellular lactate levels were measured using Amplite™ Colorimetric L-Lactate Assay Kit (AAT Bioquest) according to the manufacturer’s protocol. Briefly, 200 000 neuron progenitors were seeded and cultured on 12-well plates and cultured as normally until day 30. Then, 200 μl of the media was used for the extracellular lactate measurement and cells were harvested and lysed for the intracellular lactate measurement. Three wells per cell line were used. Extracellular samples were diluted 1:10 in PBS and all samples were filtered through a 10 kDa MW spin filter (Millipore, Darmstadt, Germany). Fluorescence was recorded at Ex/Em = A540 nm/A590 nm using an EnSpire (PerkinElmer) microplate reader and normalized to protein levels.

### Measurement of NEFL from culture medium

For measurement of NEFL, 1 ml of culture medium was collected from single 6-well neuronal cultures (four technical replicates) at day 30 of neuronal differentiation. Samples were centrifuged at 13 000 × *g* for 10 min in a cold centrifuge and supernatant frozen in −20°C. NEFL levels were quantified using the Quanterix single molecule array (Simoa, Billerica, MA, USA) HD-1 analyzer and Quanterix Simoa NF-Light Advantage Kit (ref. 103 186) according to manufacturer’s instructions. Briefly, frozen medium samples were slowly thawn on ice, mixed, and centrifuged at 10 000 × *g* for 5 min at room temperature, and diluted 1:50 in sample diluent prior to loading to a 96-well plate. Samples were measured in duplicate. All measured concentrations were within the calibration range and the mean coefficient of variation (CV) of sample replicates was 4.5%. A CV of <15% was considered acceptable between the replicates.

### Axonal regeneration capacity and mitochondrial trafficking

To evaluate axonal regeneration capacity, 100 000 neuronal progenitors were seeded on day 10 on the left chamber of a 2-compartment microfluidic device with a 450 μm microgroove barrier (XC450, XonaChip®) and cultured as normally. On day 21 axons were mechanically removed by pipetting and let them regrow for 96 h. Bright field images were taken with an EVOS M5000 Imaging System and the axons mask pixel area was determine with Fiji ImageJ. To evaluate mitochondrial axonal transport, a similar approach was followed but using a 2-compartment silicone device SND450 assembled on a glass bottom 3.5 cm dish. On day 25, neuronal media was replaced with full differentiation media supplemented with 50 nM MitoTracker™ Green FM (Thermo Fisher M22425) and cells were incubated for 1 h in the cell incubator. Then, media was replaced with full media without MitoTracker and videos were captured using an Andor Dragonfly 505 Spinning Disk Confocal microscope for 60 s, 300 ms excitation time, confocal pinhole 40 μm, objective 60× and 488 nm Ex/Em 521 nm wavelengths. Movies were taken at the most proximal region close to the grooves. Mitochondrial movements were analyzed using Fiji ImageJ plugins Difference Tracker (68) and TrackMate (69). In short, after enhancing the contrast, all movies were filtered with the Difference Tracker plugin using min diff = 3 and diff frame offset = 3. Small particles were then removed by using a median filter of 1–2 pixels. Next, moving mitochondria were extracted with a DoG detector using the TrackMate plugin of blobs with 2 μM diameter and 1 μM threshold. Movement was captured using Linear motion LAP tracker set to initial search radios = 2, search radios = 2 and max frame gaps = 2. Data were recovered using two filters: Track Displacements and Duration of Track. Data were normalized to the mitochondrial count (number of particles).

### Statistics

Significant differences between two groups were calculated with student’s *t* test or between more than two groups with One-way ANOVA followed by Tukey’s multiple comparison post-hoc test unless otherwise indicated using GraphPad Prism (GraphPad software). Mean ± standard deviation were reported in all graphs unless otherwise indicated. *P* values under 0.05 were considered statistically significant.

## Supplementary Material

Supplemental_information_ddab299
