# Supplementary material for: Threshold of heteroplasmic truncating MT-ATP6 mutation in reprogramming, Notch hyperactivation and motor neuron metabolism
Source: Hum Mol Genet. 2021 Oct 12;31(6):958–74. doi: 10.1093/hmg/ddab299 (PMC8947243; doi:10.1093/hmg/ddab299)
Supplement: Supplemental_information_ddab299 [file supplemental_information_ddab299.docx]

**SUPPLEMENTAL INFORMATION**

**Atomistic molecular dynamics simulations of mutant ATP synthase *a/c* subcomplex**

To transfer protons through F_O_ ATP synthase, the protonation state of central glutamate residues (Glu58) of c-ring must change during turnover. Consequently, we altered the protonation state of two glutamates closest to Arg159 and performed additional MD simulations. In our WT MD simulations with anionic Glu58 (in c-ring subunit c1) Arg159 orientates towards the negatively charged residue with an average Arg159 CZ – Glu58 CD distance of 4.58 ± 0.70 Å. For negatively charged Glu58 in c2 ring, Arg159 dynamically re-orientates several times between the two closest c-ring glutamates. However, in case of mutant, Arg159 is found to re-orientate towards the negatively charged C-terminus of the *a* subunit, thus reducing the Arg159 CZ – Ile209 C distance significantly, regardless of the protonation state of the c-ring glutamates (5.82 ± 1.07 Å for deprotonated Glu58 of c1, 6.34 ± 1.36 Å for deprotonated Glu58 of c2, Supplemental Figure2), and in agreement with neutral Glu58 simulations (see main text). If the premature C-terminus is protonated, longer Arg159 CZ – Glu58 CD distances are also stabilized, especially for deprotonated Glu58 of c-ring subunit c2, due to its salt-bridging with Arg159 (Figure 1 in main text). Regarding the hydration (Supplemental Figure 3), anionic character of c2 subunit Glu58 shows a similar trend as the neutral Glu58 state, thus, enhancing water occupancy in the region when C-terminus is truncated. Overall, we find that despite variation in protonation states of neighboring acidic Glu58 residues of the c-ring, the local dynamics and hydration are still different between WT and mutant enzymes.

**SUPPLEMENTAL TABLES**

**Supplemental** **Table 1.** ATP synthase model systems and simulation lengths. P (protonated) and DP (deprotonated) denote the protonation state of C-terminal of a subunit (a) and Glu58’s of the c-ring (c_1/2_).

| **System** | **Protonation State** | **Simulation Length / ns** | | |
| --- | --- | --- | --- | --- |
|  | ***a* \| c_1_ \| c_2_** | **Run 1** | **Run 2** | **Run 3** |
| **WT** | DP \| P \| P | 707 | 692 | 689 |
| **WT** | DP \| DP \| P | 406 | 620 | 444 |
| **WT** | DP \| P \| DP | 657 | 679 | 677 |
| **Mutant** | DP \| P \| P | 691 | 693 | 677 |
| **Mutant** | DP \| DP \| P | 681 | 675 | 677 |
| **Mutant** | DP \| P \| DP | 689 | 679 | 672 |
| **Mutant** | DP \| P \| P | 1281 | 678 | 660 |
| **Mutant** | DP \| DP \| P | 686 | 673 | 683 |
| **Mutant** | DP \| P \| DP | 670 | 690 | 684 |

**Supplemental Table 2. List of primers for sequencing, qPCR and fRFLP.**

| **Gene** | **Primer** | **Sequence 5’-3’** |
| --- | --- | --- |
| **Sequencing** | | |
| MT-ATP6 | Forward | CCATGGCCATCCCCTTATGA |
|  | Reverse | TGTTGTCGTGCAGGTAGAGG |
| **mtDNA copy number** | | |
| ACTB nuclear | Forward | CCTGGCACCCAGCACAAT |
|  | Reverse | GGGCCGGACTCGTCATAC |
| B2M nuclear | Forward | TGCTGTCTCCATGTTTGATGTATCT |
|  | Reverse | TCTCTGCTCCCCACCTCTAAGT |
| MT-ND5 mtDNA | Forward | AGGCGCTATCACCACTCTGTTCG |
|  | Reverse | AACCTGTGAGGAAAGGTATTCCTG |
| MT-TL1 mtDNA | Forward | CACCCAAGAACAGGGTTTGT |
|  | Reverse | TGGCCATGGGTATGTTGTTA |
| **RT-qPCR** | | |
| *CHAT* | Forward | GGAGGCGTGGAGCTCAGCGACACC |
|  | Reverse | CGGGGAGCTCGCTGACGGAGTCTG |
| *Dll1* | Forward | GATGTGATGAGCAGCATGGA |
|  | Reverse | CCATGGAGACAGCCTGGATA |
| *Hes1* | Forward | ACGACACCGGATAAACCAAA |
|  | Reverse | CGGAGGTGCTTCACTGTCAT |
| *Hes5* | Forward | AGAAAAACCGACTGCGGAAG |
|  | Reverse | GACAGCCATCTCCAGGATGT |
| *GAPDH* | Forward | CGCTCTCTGCTCCTCCTGTT |
|  | Reverse | CCATGGTGTCTGAGCGATGT |
| *Nanog* | Forward | CTCAGCCTCCAGCAGATGC |
|  | Reverse | TAGATTTCATTCTCTGGTTCTGG |
| *NEFM* | Forward | TGCAGTCCAAGAGCATCGAGC |
|  | Reverse | AGTCTCTTCACCCTCCAGGAGTT |
| *OCT4* | Forward | TTGGGCTCGAGAAGGATGTG |
|  | Reverse | TCCTCTCGTTGTGCATAGTCG |
| *Olig2* | Forward | ATGCACGACCTCAACATCGCCA |
|  | Reverse | ACCAGTCGCTTCATCTCCTCCA |
| *Sox1* | Forward | AATTTTATTTTCGGCGTTGC |
|  | Reverse | TGGGCTCTGTCTCTTAAATTTGT |
| *Sox2* | Forward | GCCCTGCAGTACAACTCCAT |
|  | Reverse | TGCCCTGCTGCGAGTAGGA |
| *TUBB3* | Forward | GCCAAGTTCTGGGAAGTCAT |
|  | Reverse | CCACTCTGACCAAAGATGAA |
| **fRFLP** |  |  |
| ATP6 | Forward | CCATGGCCATCCCCTTATGA |
|  | Reverse | GGTCATGGGCTGGGTTTTAC |
| FAM-ATP6 | FAM-Reverse | 5´-[6FAM]GGTCATGGGCTGGGTTTTAC |

**Supplemental Table 3. List of antibodies for immunocytochemistry and western blot analysis**

| **Antibody** | **Catalogue number** | **Host** | **Concentration** |
| --- | --- | --- | --- |
| **Primary** | | | |
| GFAP | R&D Syst #mab 2594 | Rabbit | 1:500 |
| CHCHD10 | Sigma #HPA003440 | Rabbit | 1:200 |
| CII-SDHA | Abcam #ab14715 | Mouse | 1:2000 WB |
| CV-ATP5 | Abcam #ab14748 | Mouse | 1:1000 WB |
| HB9 | DSHB #81.5C10-s | Mouse | 1:50 |
| ISL1 | DSHB #39.4D5-s | Mouse | 1:50 |
| MAP2 | Abcam #5392 | Chicken | 1:1000 |
| Nanog | Cell Signaling #4903 | Rabbit | 1:200 |
| NEFM | Proteintech #20664-1-AP | Rabbit | 1:800 |
| Olig2 | Millipore #AB9610 | Rabbit | 1:500 |
| PAX6 | Abcam #ab78545 | Mouse | 1:500 |
| SMA | Sigma #A2547 | Mouse | 1:500 |
| SOX1 | R&D Systems #AF1924 | Goat | 1:500 |
| TUBB3 | Sigma #T2200 | Rabbit | 1:500 |
| TUJ1 | Biolegend #801201 | Mouse | 1:1000 |
| **Secondary** | | | |
| AlexaFluor^TM^ 594 anti-rabbit IgG | A11012, Thermo Fisher |  | 1:1000 |
| AlexaFluor^TM^ 488 anti-mouse IgG | A11008, Thermo Fisher |  | 1:1000 |
| AlexaFluor^TM^ 488 anti-goat IgG | A11055, Thermo Fisher |  | 1:1000 |
| DyLight 488 anti-chicken IgG | SA5-10070, Thermo Fisher |  | 1:1000 |
| Peroxidase Goat Anti-Mouse IgG | Jackson #115-035-146 |  | 1:5000 |
| Peroxidase Goat Anti-Rabbit IgG | Molecular Probes |  | 1:5000 |
| Peroxidase Goat Anti-Goat IgG | Calbiochem |  | 1:5000 |

**SUPPLEMENTAL FIGURES**





**Supplemental Figure 1**. **Root mean square deviation (RMSD) of subunit *a* (top) and *a/c* subcomplex (bottom).** The data from WT (blue), mutant DP (red) and mutant P (green) are shown. DP – deprotonated C terminus and P – protonated C terminus, whereas C_1_ and C_2_ Glu58’s are protonated. The RMSD of backbone is calculated by aligning the trajectory to zeroth frame. Bold lines show running average of 50 simulation frames.





**Supplemental Figure 2.** **Arg159 CZ – Ile209 C distance along the individual MD simulations:** WT (top), mutant with deprotonated (DP) C-terminal (middle), and mutant with protonated (P) C-terminal (bottom). The protonated (P) and deprotonated (DP) states of C1 and C2 glutamates are mentioned on top of the plots. Bold lines show running average of 50 simulation frames.





**Supplemental Figure 3**. **Number of water molecules within 5 Å of Arg159 along the individual MD simulations:** WT (top), mutant with deprotonated (DP) C-terminal (middle), and mutant with protonated (P) C-terminal (bottom). The protonated (P) and deprotonated (DP) states of C1 and C2 glutamates are mentioned on top of the plots. Bold lines show running average of 50 simulation frames.


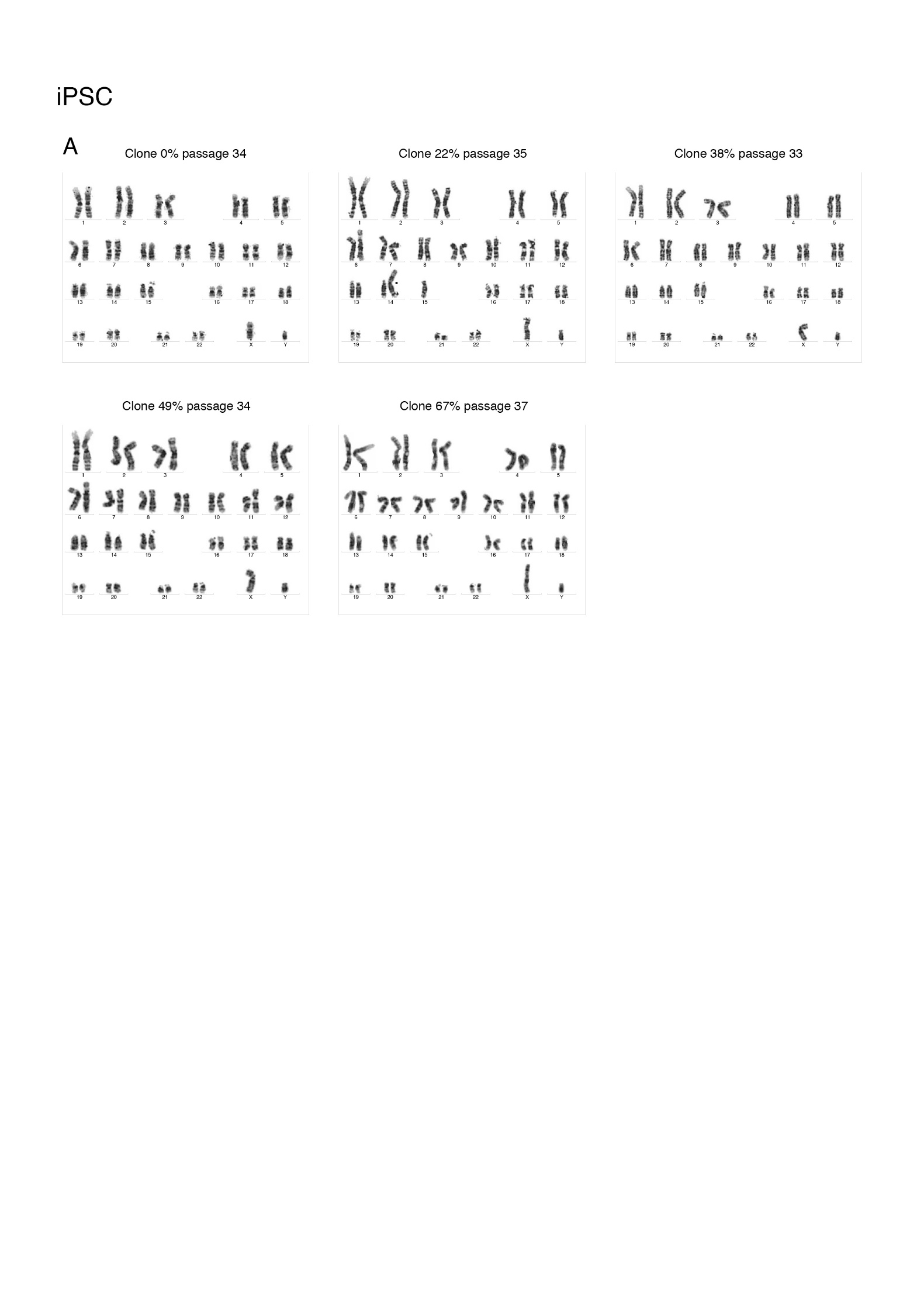


**Supplemental Figure 4. Karyotyping of patient-derived iPSC at passage ~30.** (A) Karyotype results of relevant clones utilized in the study. All show a normal karyotype with the exception of the clone 22% that harbors a translocation of the chromosome 15 into 14.

iPSC-derived motor neurons


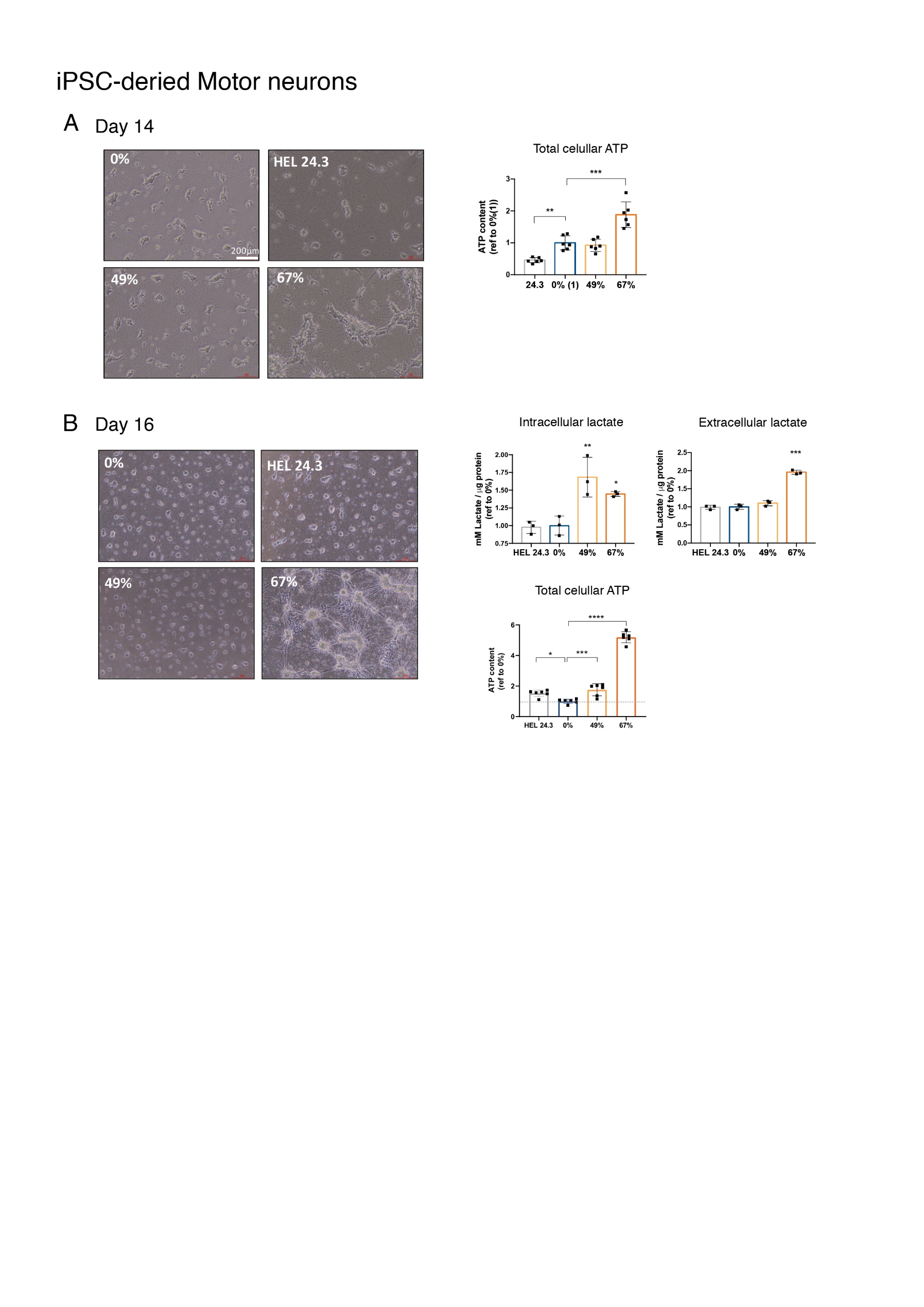


**Supplemental Figure 5. iPSC-derived motor neurons with 49% and 67% mutation load show an altered metabolism.** (A) Day 14 of the motor neuron differentiation is the earliest point when the 67% cell line shown a deficient differentiation as some cells continue dividing rather than becoming post-mitotic. Left panels, bright field microscopy images of cell lines 0%, 49%, 67% and healthy external control HEL 24.3. Note the unusual morphology of 67% colonies. Scale bar = 200µm. Right panel, Total ATP content was quantified using an ATPlite kit, n=6. (B) Day 16 of the motor neuron differentiation. Left panels, bright field microscopy images of cell lines 0%, 49%, 67% and healthy external control HEL 24.3. The phenotype of 67% cells got worst. Eventually, dividing cells cover the whole plate. Scale bar = 100µm. Right panels, Total ATP content and intracellular and extracellular lactate content were quantified using an ATPlite (n=6) and AmpLite^TM^ (n=3) kits respectively. Data shown as mean ± standard deviation; **p*<0.05, ***p*<0.01, ***p<0,001, *****p*<0.0001, by One-Way ANOVA followed by Tukey´s multiple comparison post-hoc test.

iPSC-derived motor neurons

**
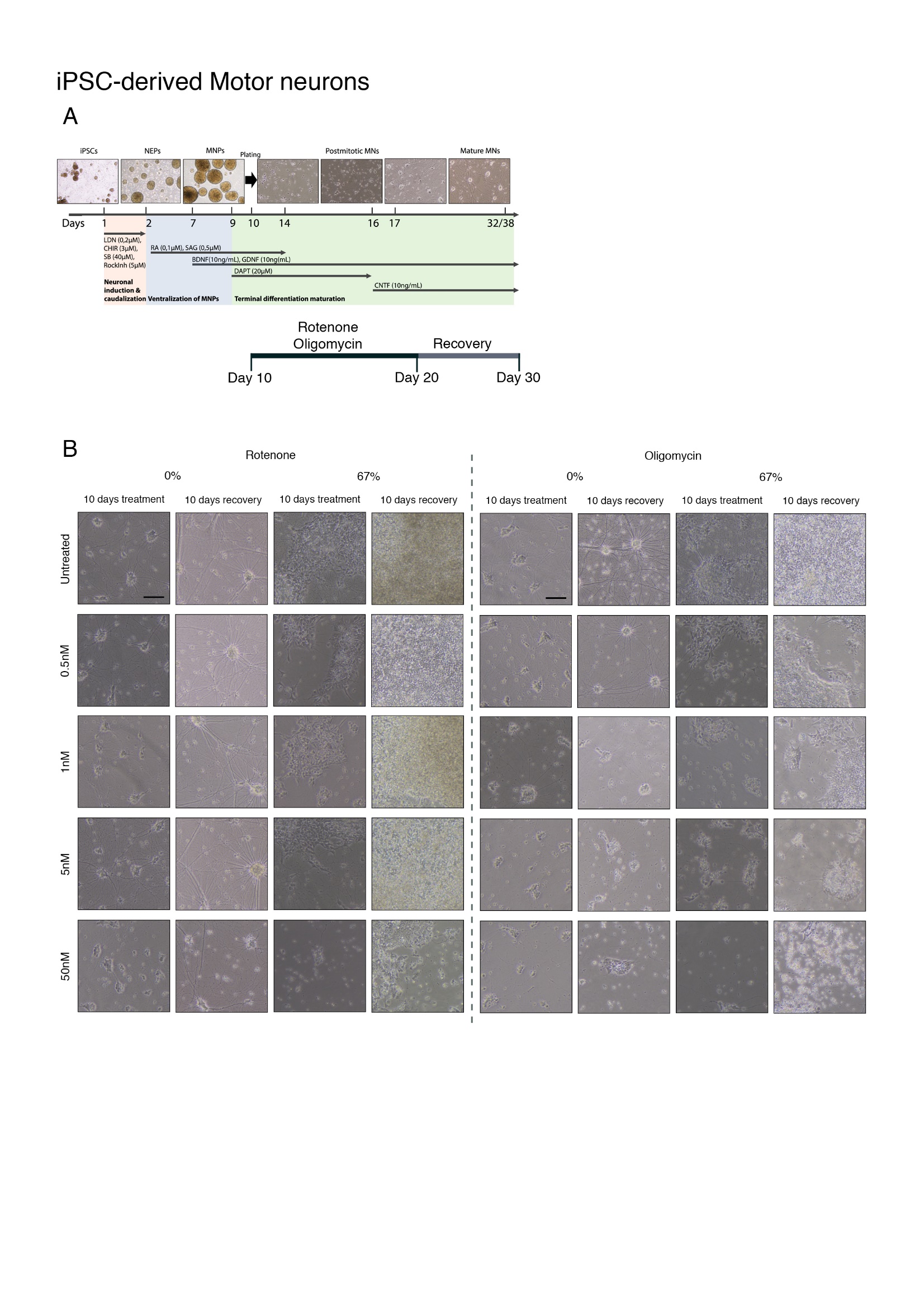
**

**Supplemental Figure 6. Mitochondrial deficiency induced by Complex I or Complex V inhibition**. (A) Schematic representation of the experimental design. Treatment with rotenone to block complex I or oligomycin to block complex V was initiated at day 10 and extended for 10 days changing the media as usual but supplemented with the mitochondrial inhibitors. At day 20, media was replaced by normal differentiation media without mitochondrial inhibitors and change every two or three days up to day 30 (10 days recovery). (B) Representative bright field microscopy images of motor neuron at day 20 (after 10 days treatment with rotenone or oligomycin with the indicated concentrations) and day 30 (after 10 days recovery) of 0% and 67% cell lines. Scale bar = 200µm. Complex V inhibition by oligomycin was more severe than rotenone as most of the cells die even with low concentrations. Still, non-lethal doses of oligomycin did not induce any sign of cell proliferation in control cells. Both treatments were especially more toxic for the mutant cells.

iPSC-derived motor neurons


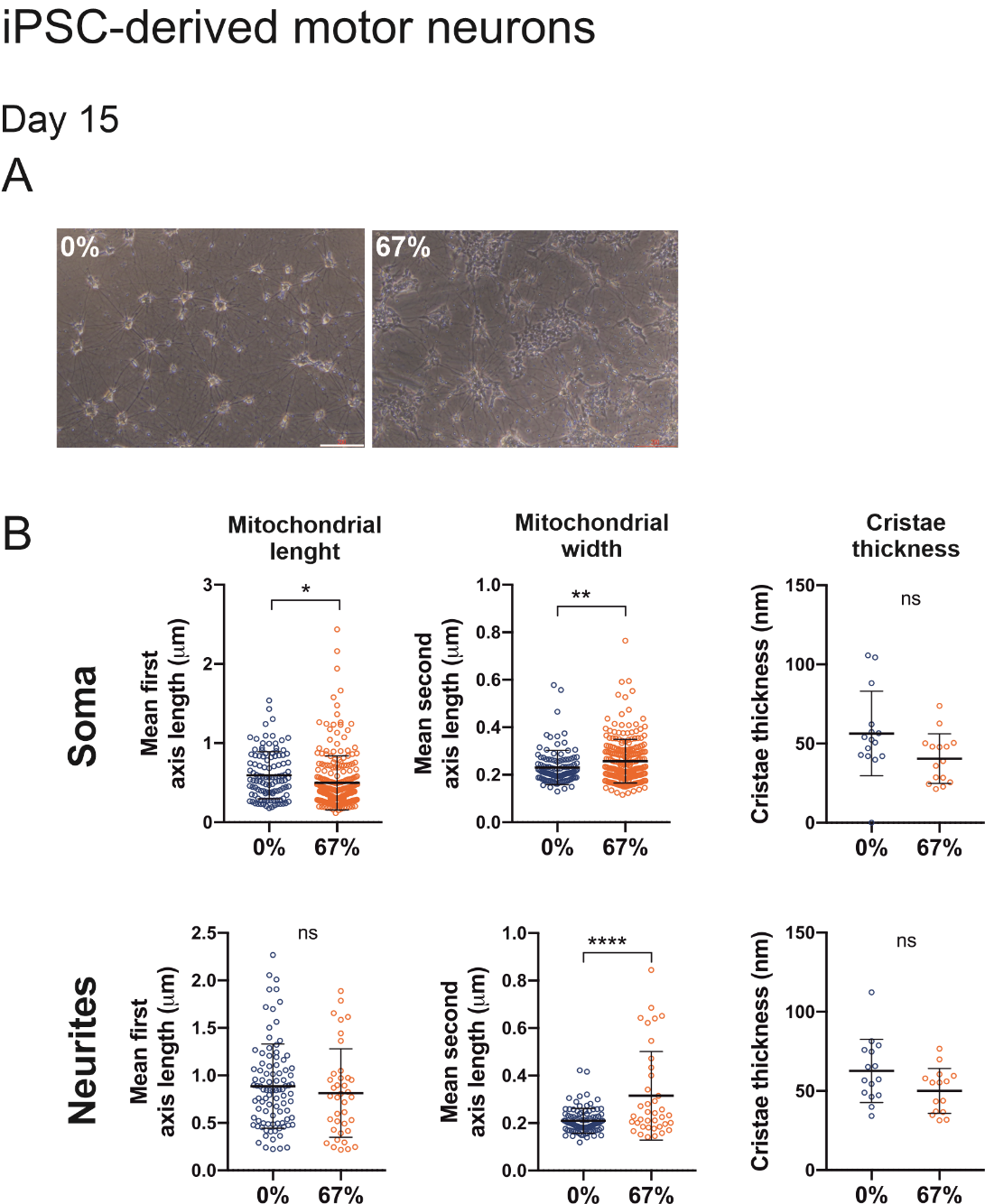


**Supplemental Figure 7. Mitochondrial ultrastructure is altered in neuron progenitors with 67% mutation load:** (A) representative bright field microscopy images of motor neuron progenitors at day 15, when the 67% mutant phenotype starts to be evident. Note that the 67% mutant cells are still able to create projections (neurites). (B) Mitochondrial morphology parameters length and width extracted from electron micrographs of somas (n=110-242 mitochondria) and neurites (n=38-94 mitochondria); and mitochondrial cristae thickness extracted from electron micrographs from somas and neurites (n=15 mitochondria). Data shown as mean ± standard deviation; ns = non-significant, **p*<0.05, ***p*<0.01, *****p*<0.0001 by Student’s *t* test.

iPSC-derived motor neurons

**
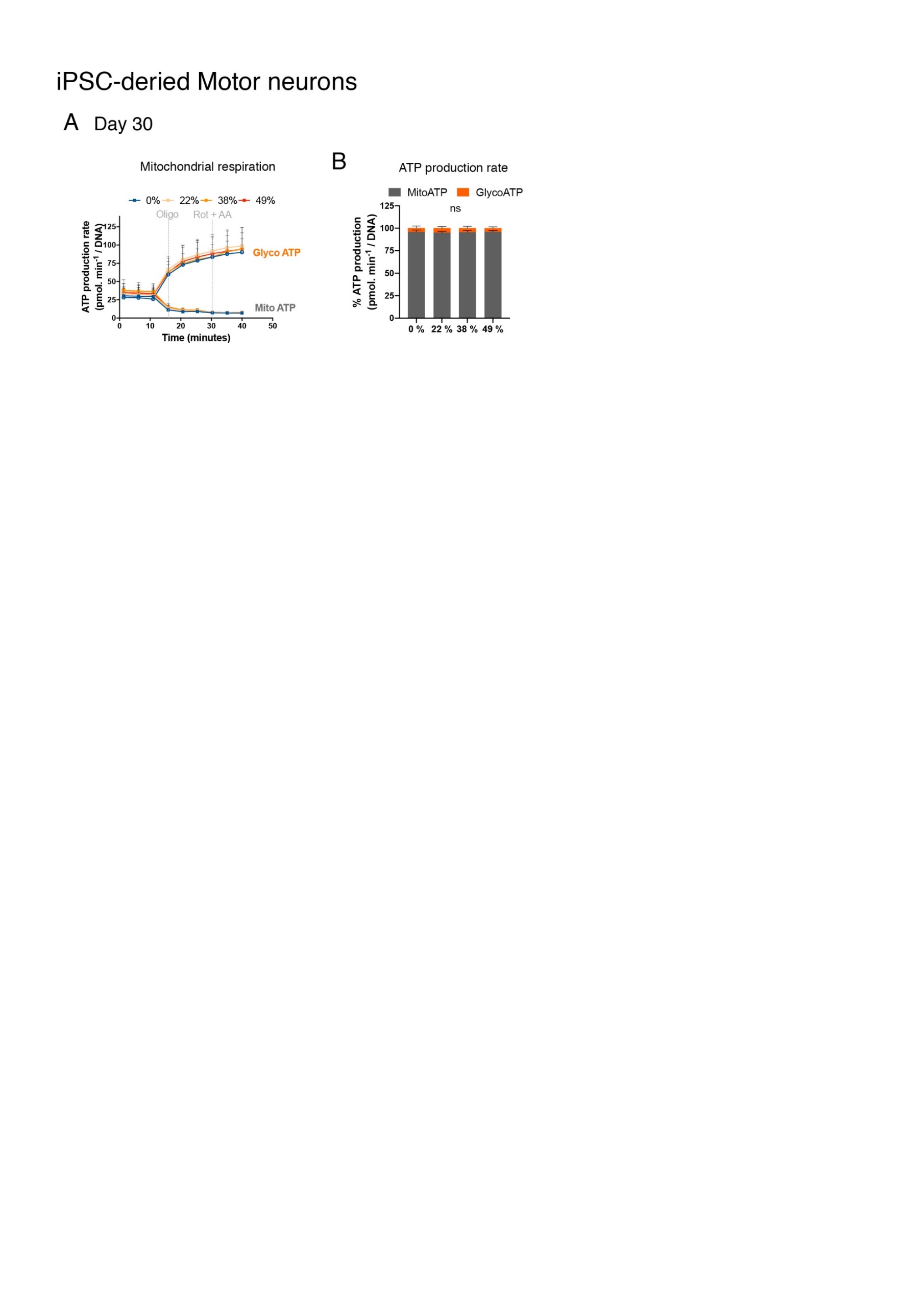
**

Day 30

A

**Supplemental Figure 8. iPSC-derived motor neurons with 49% mutation are not more glycolytic.** Day 30. Mitochondrial and glycolytic ATP production rate measured with Seahorse FX analyzer. Same data as in Figure 7B. (A) ATP production rate curves, depicting three basal respiration rate measurements followed by another three measurements after each injection (oligomycin and Rotenone + antimycin). (B) ATP production rate expressed as a % of mature MN (day 30). Data shown as mean ± standard deviation; ns, non-significant, by One-Way ANOVA followed by Dunnett’s test (versus 0%); n=15.
